# Impact of interpregnancy weight changes and perinatal outcomes: A retrospective study in Japan

**DOI:** 10.1371/journal.pone.0299794

**Published:** 2024-02-29

**Authors:** Masafumi Yamamoto, Shigeru Aoki, Satoru Shinoda, Hiroshi Ishikawa, Etsuko Miyagi

**Affiliations:** 1 Perinatal Center for Maternity and Neonate, Yokohama City University Medical Center, Yokohama, Kanagawa, Japan; 2 Department of Biostatistics, Yokohama City University School of Medicine, Yokohama, Kanagawa, Japan; 3 Department of Obstetrics and Gynecology, Yokohama City University Graduate School of Medicine, Yokohama, Japan; 4 Department of Obstetrics and Gynecology, Kanagawa Children’s Medical Center, Yokohama, Kanagawa, Japan; Mount Sinai Health System, University of Toronto, CANADA

## Abstract

Previous studies have shown that interpregnancy weight fluctuations impact perinatal outcomes. In order to examine this in Japanese women, we analyzed the data of 2,861 women in their first and second pregnancies who delivered singletons between 2000 and 2022. We compared the second pregnancy perinatal outcomes of women whose interpregnancy body mass index (BMI) change was -1 to 1 unit with those of women whose BMI change was < -1 or ≥ 1 unit. An interpregnancy BMI change ≥ 1 unit was associated with an increased risk of developing gestational diabetes mellitus (adjusted odds ratio [aOR], 1.51; 95% confidence interval [CI], 1.18–1.95) and delivering a large for gestational age neonate (aOR, 1.67; 95% CI, 1.15–2.42) but a decreased risk of preterm birth (aOR, 0.66; 95% CI, 0.46–0.95). An interpregnancy BMI change < -1 unit was associated with a decreased risk of developing gestational diabetes mellitus (aOR, 0.51; 95% CI, 0.31–0.85). In a subgroup analysis of three groups divided according to prepregnancy BMI, interpregnancy BMI changes ≥ 1 unit in women with a BMI of < 18.5 kg/m2 before their first pregnancy were associated with a remarkable risk reduction of developing preterm birth (aOR, 0.30; 95% CI, 0.11–0.81). Interpregnancy BMI changes < -1 unit in women with a BMI of ≥ 25 kg/m^2^ before their first pregnancy were associated with a remarkable risk reduction of developing gestational diabetes mellitus (aOR, 0.33; 95% CI, 0.12–0.88). Weight gain during interpregnancy period was related to an increased risk of gestational diabetes mellitus and delivery of a large-for-gestational-age neonate, whereas weight loss was related to a decreased risk of developing gestational diabetes mellitus. These results indicate the importance of interpregnancy weight control as part of preconception care; therefore, women considering additional pregnancies should be educated on the importance of maintaining a healthy weight.

## Introduction

Previous studies have shown that weight changes occurring in the interim between the first and second pregnancy affect the outcomes of the second and subsequent pregnancies. A large study in Sweden showed that an interpregnancy increase in body mass index (BMI) ≥ 3 units between the first and second pregnancy increased the risk of developing hypertensive disorders of pregnancy (HDP), gestational diabetes mellitus (GDM), and delivery of a large for gestational age (LGA) neonate [1]. A retrospective cohort study in Belgium showed an increased risk of developing GDM and HDP in subsequent pregnancies among normal-weight women with a BMI change of ≥ 2 units during the interpregnancy period [2]. A recently published systematic review also found that excessive weight gain during the interpregnancy period increased the risk of HDP, GDM, and delivery of LGA neonates [3–6].

However, these studies were conducted on European and American populations, and insufficient research has been conducted in Japanese women. It is known that many Japanese women are relatively underweight [7], and it is uncertain whether the results of these previous studies apply to Japanese women as well. Therefore, this study aimed to examine the effect of interpregnancy weight changes on the perinatal outcomes of subsequent pregnancies in Japanese women.

## Materials and methods

### Study design

**Fig 1:**
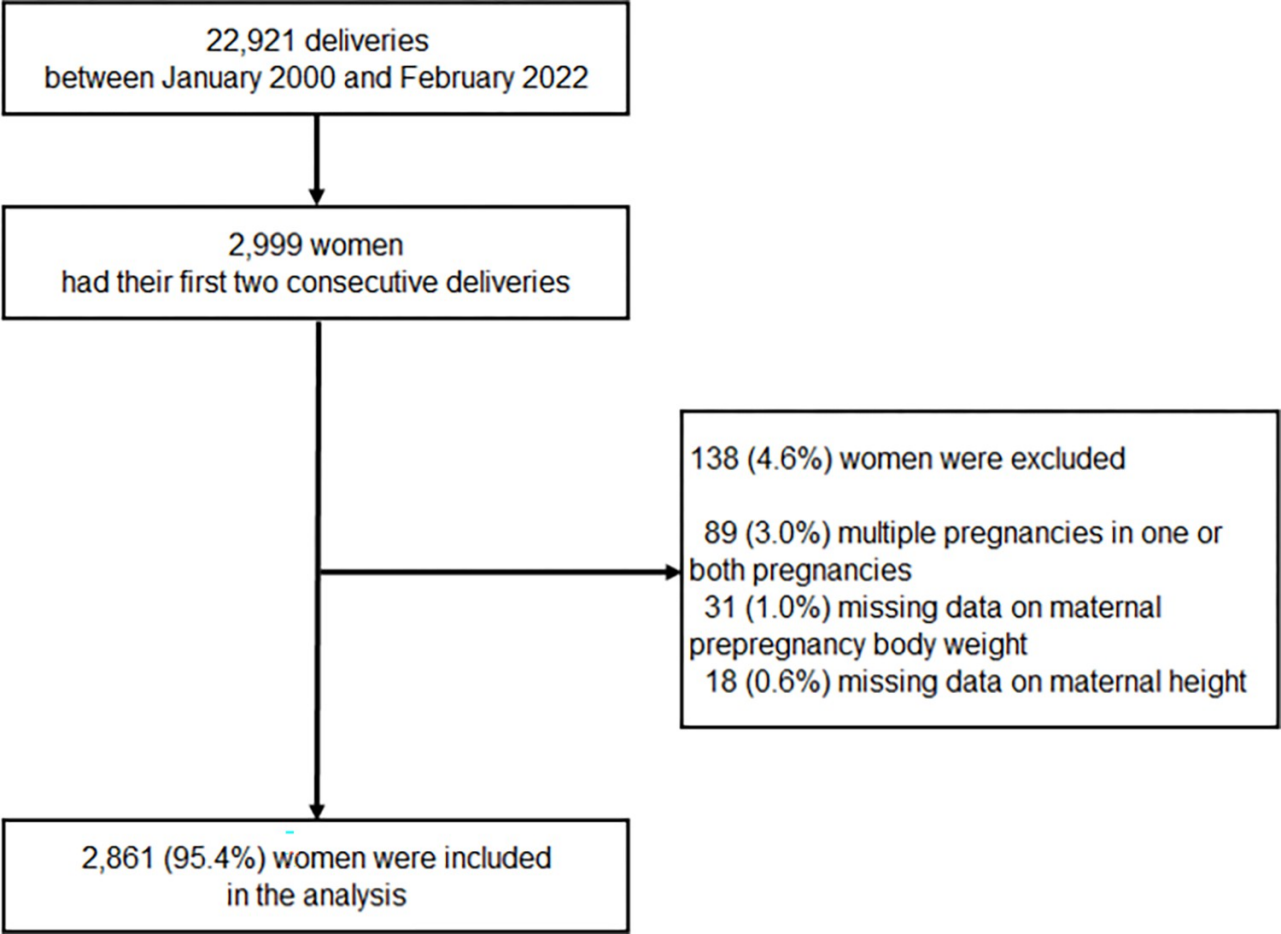
Flowchart in the selection of study participants.

This retrospective study was conducted using medical record data of women who delivered at Yokohama City University Medical Center, Yokohama, Kanagawa Prefecture, Japan, between January 1, 2000, and February 28, 2022. Among the 22,921 deliveries managed in this period, 5,998 were in 2,999 women whose first and second deliveries were both managed at our hospital. The participants included in our study were those who had two singleton subsequent pregnancies (their first and second pregnancies). Participants were excluded if their first or second pregnancy was with multiples or if their prepregnancy weight or height information was missing. Based on these criteria, 2,861 women were included in the analysis (Fig 1).

This study was conducted between June 14, 2022, and July 28, 2023, after approval by the Yokohama City University Ethics Committee (approval number: F220400012). Due to the retrospective nature of the study, the Ethics Committee waived the requirement of written informed consent from the participants. All procedures were performed in accordance with the Declaration of Helsinki and with relevant guidelines and regulations. This study was reported according to the (S1 Checklist) STROBE statement [8].

### Data collection and outcomes

Data on the participants’ first and second pregnancies were collected from the medical records: age, height, prepregnancy weight, presence of HDP, presence of GDM, weight at delivery, gestational age at delivery, and height and weight of the neonate. Prepregnancy BMI was calculated based on prepregnancy weight and height. The change in the interpregnancy BMI was calculated by subtracting the value of the first prepregnancy BMI from the prepregnancy BMI at the second pregnancy. The interpregnancy interval was defined as the period between the first delivery date and the estimated conception date in the second pregnancy. The estimated conception date was calculated from estimated due date of the participants. HDP was defined as a systolic blood pressure ≥ 140 mmHg or diastolic blood pressure ≥ 90 mmHg occurring multiple times during the gestational period, applying the definition of Japan Society for the study of Hypertension in Pregnancy [9]. GDM was defined, according to the guidelines of the Japan Society of Obstetrics and Gynecology [10], as meeting one or more of the following criteria regarding blood glucose levels in a 75-g oral glucose tolerance test performed during pregnancy: fasting ≥ 92 mg/dL, 1 h after load ≥ 180 mg/dL, and 2 h after load ≥ 153 mg/dL. In Japan, the former criteria were applied before 2010; however, in the present study, GDM was defined by applying the above criteria to participants before 2010. Preterm birth was defined as delivery from 22 weeks 0 days to 36 weeks 6 days of gestation. Neonates whose birth weight was less than the 10th percentile for gestational age were defined as small for gestational age (SGA) and those whose birth weight was greater than the 90th percentile were defined as LGA. The definitions of SGA and LGA were based on Japanese neonatal anthropometric charts presented by the Japan Pediatric Society [11].

The change in the interpregnancy BMI was categorized into < -1, -1 to < 1, and ≥ 1 unit (kg/m^2^). The primary endpoint was the risk of developing HDP, GDM, preterm birth, and delivery of LGA and SGA infants; the risk was compared to other categories using interpregnancy BMI changes -1 to < 1 unit as a control. Since the outcome needed to be interpreted in relation to the BMI at the time of the first pregnancy, participants were stratified into three groups according to their prepregnancy BMI at the time of the first pregnancy according to the Japan Society for the Study of Obesity criteria [12]: underweight (< 18.5 kg/m^2^), normal-weight (18.5–24.9 kg/m^2^), and obese (≥ 25.0 kg/m^2^). Additionally, an analysis of the perinatal outcomes in each group was performed.

### Statistical analysis

As the main study analysis, multiple logistic regression analysis was performed to calculate adjusted odds ratios for each endpoint by the change in interpregnancy BMI. The covariates were maternal age and BMI before the first pregnancy, interpregnancy interval, and gestational weight gain in second pregnancy. The same analysis was repeated for each subgroup. Data were presented as the median and interquartile range for continuous variables, and as numbers and proportions for categorical variables. All analyses were performed using JMP Pro 16.0 software (SAS Institute, Cary, NC, USA).

## Results

Table 1 shows the maternal characteristics, data on the interpregnancy period, and first and second pregnancy perinatal outcomes. The median interval between the first and second pregnancies was 33 months. The second prepregnancy weight was a median of 1.0 kg (0.4 BMI units) higher than the first pregnancy weight. In the second pregnancy, the frequency of preterm birth, HDP, and delivery of LGA infants decreased, whereas the frequency of GDM and delivery of SGA infants increased.

**Table 1:** Maternal characteristics and perinatal outcomes at the first and second pregnancy.

	First pregnancy	Second pregnancy
Age (years)	30.0 (27.0–33.0)	33.0 (30.0–36.0)
Height (cm)	158.0 (155–162)	158.0 (155–162)
Prepregnancy weight (kg)	51.0 (47.0–56.0)	52.0 (48.0–57.6)
Prepregnancy BMI (kg/m^2^)	20.2 (19.0–22.0)	20.6 (19.1–22.8)
Gestational weight gain (kg)	10.0 (7.3–12.5)	9.6 (7.1–11.9)
Gestational week at delivery (week)	39.5 (38.4–40.4)	39.0 (38.1–39.9)
Preterm birth (%)	260 (9.1)	188 (6.6)
Hypertensive disorders of pregnancy (%)	137 (4.8)	104 (3.6)
Gestational diabetes (%)	227 (7.9)	347 (12.1)
Large for gestational age (%)	225 (7.9)	138 (4.8)
Small for gestational age (%)	253 (8.8)	346 (12.1)
Interpregnancy interval (months)		33.4 (25.8–45.6)
Interpregnancy weight change (kg)		1.0 (-1.0–3.0)
Interpregnancy BMI change (kg)		0.38 (-0.3–1.2)

Data are presented as median (interquartile range); BMI: body mass index

Table 2 shows the number of participants stratified according to the change in interpregnancy BMI, and maternal characteristics and perinatal outcomes at first pregnancy. The interpregnancy BMI changes differed by age: the largest percentage of participants with a change in interpregnancy BMI of < -1 unit (28.3%) was observed among women who were aged ≤ 19 years during their first pregnancy. This age group also had the smallest percentage of participants with > 1 unit change in interpregnancy BMI (17.4%). Women aged 25 to 29 years during their first pregnancy showed the lowest percentage of participants with an interpregnancy BMI change < -1 unit (8.4%) but the highest percentage with ≥ 1 unit change in interpregnancy BMI (33.3%). Except for the interpregnancy interval ≤ 11 months, the proportion of participants with interpregnancy BMI changes < -1 unit tended to decrease, whereas that of participants with interpregnancy BMI changes ≥ 1 unit tended to increase, as the interpregnancy interval increased. From the aspect of first prepregnancy BMI, there was a smaller proportion of participants with interpregnancy BMI changes < -1 unit among women with a first prepregnancy BMI < 18.5 kg/m^2^. On the other hand, there was a smaller proportion of participants with an interpregnancy BMI change of -1 to 1 unit and a larger proportion of those with an interpregnancy BMI change of < -1 and ≥ 1 unit among women with a first prepregnancy BMI ≥ 25 kg/m^2^. Perinatal morbidity in the first pregnancy were not found to affect changes in interpregnancy BMI.

**Table 2:** Participants classified by interpregnancy BMI changes and maternal characteristics and perinatal outcomes at first pregnancy.

		Interpregnancy BMI change category		
		< -1	-1 to < 1	≥ 1
Age (years)				
	≤ 19	13/46 (28.3)	25/46 (54.3)	8/46 (17.4)
	20–24	37/287 (12.9)	159/287 (55.4)	91/287 (31.7)
	25–29	84/1000 (8.4)	583/1000 (58.3)	333/1000 (33.3)
	30–34	112/1073 (10.4)	686/1073 (63.9)	275/1073 (25.6)
	35–39	47/424 (11.1)	253/424 (59.7)	124/424 (29.2)
	≥ 40	3/31 (9.7)	20/31 (64.5)	8/31 (25.8)
Prepregnancy BMI (kg/m^2^)				
	< 18.5	17/489 (3.5)	330/489 (67.5)	142/489 (29.0)
	18.5–24.9	218/2134 (10.2)	1322/2134 (61.9)	594/2134 (27.8)
	≥ 25.0	61/238 (25.6)	74/238 (31.1)	103/238 (43.3)
Interpregnancy interval (months)				
	≤ 11	1/11 (9.1)	4/11 (36.4)	6/11 (54.5)
	12–23	76/515 (14.8)	313/515 (60.8)	126/515 (24.5)
	24–35	121/1104 (11.0)	717/1104 (64.9)	266/1104 (24.1)
	36–47	49/546 (9.0)	348/546 (63.7)	149/546 (27.3)
	≥ 48	49/685 (7.2)	344/685 (50.2)	292/685 (42.6)
Preterm birth				
	No	261/2601 (10.0)	1583/2601 (60.9)	757/2601 (29.1)
	Yes	35/260 (13.5)	143/260 (55.0)	82/260 (31.5)
Hypertensive disorders of pregnancy				
	No	280/2724 (10.3)	1651/2724 (60.6)	793/2724 (29.1)
	Yes	16/137 (11.7)	75/137 (54.7)	46/137 (33.6)
Gestational diabetes				
	No	261/2634 (9.9)	1601/2634 (60.8)	772/2634 (29.3)
	Yes	35/227 (15.4)	125/227 (55.1)	67/227 (29.5)
Large for gestational age				
	No	272/2636 (10.3)	1598/2636 (60.6)	766/2636 (29.1)
	Yes	24/225 (10.7)	128/225 (56.9)	73/225 (32.4)
Small for gestational age				
	No	266/2608 (10.2)	1585/2608 (60.8)	757/2608 (29.0)
	Yes	30/253 (11.9)	141/253 (55.7)	82/253 (32.4)

% in brackets; BMI, body mass index

Table 3 shows the adjusted odds ratios for each interpregnancy BMI change category. A BMI increase of ≥ 1 unit between pregnancies increased the risk of developing GDM (adjusted odds ratio [aOR], 1.51; 95% confidence interval [CI], 1.18–1.95), whereas a decrease of ≥ 1 unit decreased this risk (aOR, 0.51; 95% CI, 0.31–0.85). Moreover, an increase in BMI of > 1 unit raised the risk of delivering an LGA neonate (aOR, 1.67; 95% CI, 1.15–2.42) but lowered the risk of preterm birth (aOR, 0.66; 95% CI, 0.46–0.95). Although there was no statistically significant difference in the risk of developing HDP and delivering SGA neonates, it was observed that weight gain tended to increase the risk of developing HDP, whereas weight loss tended to increase the risk of delivering SGA neonates.

**Table 3:** Adjusted odds ratios for adverse perinatal outcomes during the second pregnancy in relation to interpregnancy BMI changes.

Outcomes (n)	Interpregnancy BMI change (kg/m^2^)	n (%)	adjusted odds ratio*	95% CI
GDM	< -1	21 (7.1)	0.51	0.31–0.85
(n = 347)	-1 to < 1 (reference)	175 (10.1)	1.00	
	≥ 1	151 (18.0)	1.53	1.18–1.97
HDP	< -1	11 (3.7)	0.86	0.43–1.75
(n = 104)	-1 to < 1 (reference)	50 (2.9)	1.00	
	≥ 1	43 (5.1)	1.39	0.89–2.17
LGA	< -1	12 (4.1)	0.61	0.32–1.19
(n = 138)	-1 to < 1 (reference)	71 (4.1)	1.00	
	≥ 1	55 (6.6)	1.62	1.11–2.36
SGA	< -1	40 (13.5)	1.43	0.97–2.09
(n = 346)	-1 to < 1 (reference)	213 (12.3)	1.00	
	≥ 1	93 (11.1)	0.77	0.58–1.01
Preterm birth	< -1	19 (6.4)	1.13	0.66–1.91
(n = 188)	-1 to < 1 (reference)	118 (6.8)	1.00	
	≥ 1	51 (6.1)	0.66	0.46–0.95

BMI, body mass index; CI, confidence interval; GDM, gestational diabetes; HDP, hypertensive disorders of pregnancy; LGA, large for gestational age; SGA, small for gestational age

*Adjusted for maternal age, maternal BMI before first pregnancy, interpregnancy interval, and gestational weight gain in second pregnancy.

Table 4 shows the adjusted odds ratios for the endpoints of each interpregnancy BMI change category stratified by the prepregnancy BMI. Among underweight women, a BMI gain of ≥ 1 unit between pregnancies increased the risk of developing GDM (aOR, 2.14; 95% CI, 1.10–4.16) but decreased the risk of preterm birth (aOR, 0.30; 95% CI, 0.11–0.81). Among normal-weight women, an interpregnancy BMI gain of ≥ 1 unit increased the risk of developing GDM (aOR, 1.52; 95% CI, 1.13–2.04) and delivering an LGA neonate (aOR, 1.81; 95% CI, 1.19–2.76), respectively. Among obese women, a BMI decrease of ≥ 1 unit significantly decreased the risk of developing GDM (aOR, 0.33; 95% CI, 0.12–0.88). Other endpoints were not significantly associated with interpregnancy BMI changes.

**Table 4:** Adjusted odds ratios for adverse perinatal outcomes during the second pregnancy in relation to interpregnancy BMI changes between the first and second pregnancy stratified by prepregnancy BMI at the first pregnancy.

Outcomes	Interpregnancy BMI change (kg/m^2^)	Underweight (BMI < 18.5) n = 489		Normal weight (BMI 18.5–2.49) n = 2134		Overweight (BMI ≥ 25) n = 238	
		n (%)	aOR* (95% CI)	n (%)	aOR* (95% CI)	n (%)	aOR* (95% CI)
GDM	< -1	0	NA	14 (6.4)	0.71	7 (11.5)	0.33
					(0.40–1.27)		(0.12–0.89)
	-1 to < 1 (reference)	22 (6.7)	1.00	133 (10.1)	1.00	20 (27.0)	1.00
	≥ 1	19 (13.4)	2.05	94 (15.8)	1.56	38 (36.9)	1.12
			(1.04–4.03)		(1.16–2.10)		(0.55–2.28)
HDP	< -1	1 (5.9)	3.07	3 (1.4)	0.53	7 (11.5)	1.18
			(0.33–28.4)		(0.16–1.76)		(0.39–3.56)
	-1 to < 1 (reference)	6 (1.8)	1.00	36 (2.7)	1.00	8 (10.8)	1.00
	≥ 1	2 (1.4)	0.71	26 (4.4)	1.49	15 (14.6)	1.35
			(0.13–3.77)		(0.87–2.52)		(0.51–3.61)
LGA	< -1	0	NA	7 (3.2)	0.67	5 (8.2)	0.96
					(0.30–1.50)		(0.28–3.35)
	-1 to < 1 (reference)	9 (2.7)	1.00	56 (4.2)	1.00	6 (8.1)	1.00
	≥ 1	6 (4.2)	1.64	40 (6.7)	1.77	9 (8.7)	1.27
			(0.53–5.08)		(1.15–2.71)		(0.41–3.95)
SGA	< -1	2 (11.8)	0.67	30 (13.8)	1.36	8 (13.1)	1.83
			(0.15–3.10)		(0.89–2.09)		(0.57–5.92)
	-1 to < 1 (reference)	53 (16.1)	1.00	154 (11.6)	1.00	6 (8.1)	1.00
	≥ 1	20 (14.1)	0.78	63 (10.6)	0.74	10 (9.7)	0.73
			(0.44–1.38)		(0.54–1.02)		(0.23–2.31)
Preterm birth	< -1	3 (17.7)	2.12	14 (6.4)	1.26	2 (3.3)	0.41
			(0.55–8.20)		(0.69–2.30)		(0.07–2.29)
	-1 to < 1 (reference)	30 (9.0)	1.00	83 (6.3)	1.00	5 (6.8)	1.00
	≥ 1	5 (3.5)	0.30	38 (6.4)	0.73	8 (7.8)	1.00
			(0.11–0.81)		(0.48–1.11)		(0.29–3.44)

BMI, body mass index; aOR, adjusted odds ratio; CI, confidence interval; NA, not applicable; GDM, gestational diabetes; HDP, hypertensive disorders of pregnancy; LGA, large for gestational age; SGA, small for gestational age

*Adjusted for maternal age, interpregnancy interval, and maternal gestational weight gain in second pregnancy.

## Discussion

This retrospective study suggested that a BMI gain of ≥ 1 unit between the first and second pregnancy increased the risk of developing GDM and delivering an LGA neonate but decreased the risk of preterm birth in the subsequent pregnancy. In addition, the study indicated that a BMI loss of ≥ 1 unit between the first and second pregnancy decreased the risk of developing GDM in the second pregnancy.

A BMI gain of > 1 unit between the first and second pregnancy was associated with an increased risk of developing GDM and delivering an LGA neonate during the second pregnancy. The relations between the degree of change in the BMI and the risk of GDM and delivering LGA infants varied according to previous studies. Ehrlich et al. [13] and Lynes et al. [14], who studied Americans, Sorbye et al. [15], who studied a Norwegian population, and Villamor et al. [1], who studied a Swedish population, reported a significantly increased risk of developing GDM in subsequent pregnancies with a BMI increase of ≥ 1 unit, which is consistent with our findings. Sorbye et al. [16], who studied Norwegian and Swedish populations, reported a significantly increased risk of developing GDM with a BMI gain of ≥ 2 units, and Bogaerts et al. [2], who studied a Belgian population, reported a significantly increased risk of developing GDM with a ≥ 3-unit gain in BMI. In addition, the above-mentioned study by Villamor et al. [1] showed a significant increase in the risk of delivering an LGA neonate in subsequent pregnancies with a BMI gain of ≥ 1 unit. Benjamin et al. [17] conducted a study in Americans and reported a significant increase in the risk of delivering an LGA neonate with a BMI gain of ≥ 2 units. Although there are racial differences in the prevalence of GDM [18, 19], the current study on Japanese women indicated that weight gain between pregnancies is a risk factor for developing GDM and delivering an LGA neonate in the second pregnancy, and that the increased risk occurs with as little as a 1-unit increase in BMI. In contrast, a BMI gain of > 1 unit between the first and second pregnancy was associated with a decreased risk of preterm birth in the subsequent pregnancy. In the subgroup analysis, an interpregnancy BMI gain of > 1 unit was associated with a decreased risk of preterm birth only in the underweight group. In the systematic review by Nagpal et al. [6], the authors reported that there were no significant associations between interpregnancy BMI changes and preterm birth. Previous studies found that women who were underweight (BMI < 18.5 kg/m^2^) before pregnancy were associated with an increased risk of preterm birth [20, 21]. Japanese women are relatively more likely to be underweight; in a Japanese survey, 21.8% of women in the 20–29 age group and 17.1% of women in the 30–39 age group were underweight [7]. In the current study, 17.1% (489 of 2861) women were underweight. Compared to previous studies, which were mainly conducted on European and American populations, an increase in interpregnancy BMI may have resulted in a decrease in the preterm birth because Japanese women are more likely to be underweight.

A BMI loss of ≥ 1 units between the first and second pregnancy was associated with a decreased risk of developing GDM in the second pregnancy. In an American study, Ehrlich et al. [13] reported that women with a BMI ≥ 25 kg/m^2^ before their first pregnancy had a significantly reduced risk of developing GDM in their second pregnancy if their interpregnancy BMI decreased by 1–2 units. In this study, the same degree of weight loss resulted in a decreased risk of GDM in subsequent pregnancies. Previous studies indicated that insulin sensitivity improves in obese individuals when they lose weight [22]; thus, improved insulin sensitivity as a result of losing weight during the interpregnancy period may have contributed to these results.

The subgroup analysis results showed that women with a BMI of ≥ 25 kg/m^2^ before their first pregnancy had a one-third reduced risk of developing GDM in their subsequent pregnancy if their BMI decreased by at least 1 unit during the interpregnancy period. Previous studies have shown the effectiveness of weight loss in obese women. Glazer et al. [23] studied the relations between interpregnancy weight change and the risk of GDM in obese women and reported that weight loss significantly reduced the risk of developing GDM in the subsequent pregnancy. In 2006, the Center for Disease Control and Prevention in the United States proposed preconception care, which recommends that obese women lose weight before pregnancy [24]. This study indicated that weight reduction in obese women significantly decreased the risk of GDM in the following pregnancy, thereby highlighting the importance of preconception care. In previous studies, diet, exercise [25], and breastfeeding [26, 27] have been reported to be effective factors in promoting postpartum weight loss. Therefore, obstetricians need to provide postpartum women with the results of the current and previous studies in order to educate them on the importance of postpartum weight management as preconception care.

One strength of this study is that it is one of the few studies to examine the relations between interpregnancy weight changes and perinatal outcomes in Japanese women. Although studies investigating the relations between interpregnancy BMI changes and HDP or GDM in Japanese women were recently published [28, 29], to our knowledge this is the first study to examine interpregnancy weight fluctuations and LGA or SGA in neonates. However, because this was a single-center retrospective observational study, there were some limitations. First, although the single-center nature of this study allowed us to examine the medical records of participants in detail, it is possible that our study participants were a higher-risk group of women regarding pregnancy compared to the general population because our hospital is a tertiary care facility. Second, because this was a single-center study, the number of enrolled participants was limited. This study showed significant differences between interpregnancy weight changes and the risk of developing GDM and delivering LGA neonates; however, in contrast to previous studies, it did not show significant associations between the risk of developing HDP and delivering SGA neonates. It may be possible to demonstrate statistically significant differences by increasing the number of participants. In addition, there are other possible confounding factors that should be adjusted in the analysis. However, we had to narrow the number of covariates in the regression analysis because this study was conducted at a single center and included a limited number of cases and outcome events. Third, due to the retrospective nature of this study, we were unable to obtain some information from the medical records, such as ethnic or migrant background and socioeconomic status of the participants. A multicenter prospective study is required to address these issues.

In conclusion, this study suggested that an interpregnancy BMI gain of ≥ 1 unit increased the risk of developing GDM and delivering an LGA neonate but decreased the risk of preterm birth especially in underweight women, and an interpregnancy BMI loss of ≥ 1 unit decreased the risk of developing GDM in the second pregnancy. The results of the current study further underscore the importance of prenatal care; therefore, obstetricians should inform women who are considering a second pregnancy on the importance of weight management as part of preconception care.

## Supporting information

S1 Checklist STROBE statement—checklist of items that should be included in reports of observational studies. (DOCX)
